# The impact of placental genomic risk for schizophrenia and birth asphyxia on brain development

**DOI:** 10.1038/s41398-023-02639-4

**Published:** 2023-11-08

**Authors:** Laura A. Wortinger, Alexey A. Shadrin, Attila Szabo, Stener Nerland, Runar Elle Smelror, Kjetil Nordbø Jørgensen, Claudia Barth, Dimitrios Andreou, Marianne Thoresen, Ole A. Andreassen, Srdjan Djurovic, Gianluca Ursini, Ingrid Agartz

**Affiliations:** 1 Department of Psychiatric Research, Diakonhjemmet Hospital, Oslo, Norway; 2 NORMENT, Institute of Clinical Medicine, University of Oslo, Oslo, Norway; 3 NORMENT, Division of Mental Health and Addiction, Oslo University Hospital, Oslo, Norway; 4 KG Jebsen Centre for Neurodevelopmental Disorders, University of Oslo, Oslo, Norway; 5 Department of Psychiatry, Telemark Hospital, Skien, Norway; 6 Centre for Psychiatry Research, Department of Clinical Neuroscience, Karolinska Institutet and Stockholm Health Care Services, Stockholm County Council, Stockholm, Sweden; 7 Department of Physiology, Institute of Basic Medical Sciences, University of Oslo, Oslo, Norway; 8 Neonatal Neuroscience, Translational Health Sciences, University of Bristol, Bristol, United Kingdom; 9 Department of Medical Genetics, Oslo University Hospital, Oslo, Norway; 10 NORMENT, Department of Clinical Science, University of Bergen, Bergen, Norway; 11 Lieber Institute for Brain Development, Johns Hopkins Medical Campus, Baltimore, MD USA; 12 Department of Psychiatry and Behavioral Sciences, Johns Hopkins University School of Medicine, Baltimore, MD USA

**Keywords:** Personalized medicine, Molecular neuroscience

## Abstract

The placenta plays a role in fetal brain development, and pregnancy and birth complications can be signs of placental dysfunction. Birth asphyxia is associated with smaller head size and higher risk of developing schizophrenia (SZ), but whether birth asphyxia and placental genomic risk factors associated with SZ are related and how they might impact brain development is unclear. 433 adult patients with SZ and 870 healthy controls were clinically evaluated and underwent brain magnetic resonance imaging. Pregnancy and birth information were obtained from the Medical Birth Registry of Norway. Polygenic risk scores (PRS) from the latest genome-wide association study in SZ were differentiated into placental PRS (PlacPRS) and non-placental PRS. If the interaction between PRSs and birth asphyxia on case-control status was significant, neonatal head circumference (nHC) and adult intracranial volume (ICV) were further evaluated with these variables using multiple regression. PlacPRS in individuals with a history of birth asphyxia was associated with a higher likelihood of being a patient with SZ (*t* = 2.10, *p* = 0.018). We found a significant interaction between PlacPRS and birth asphyxia on nHC in the whole sample (*t* = −2.43, *p* = 0.008), with higher placental PRS for SZ associated with lower nHC in those with birth asphyxia. This relationship was specific to males (*t* = −2.71, *p* = 0.005) and also found with their adult ICV (*t* = −1.97, *p* = 0.028). These findings suggest that placental pathophysiology and birth asphyxia may affect early and late trajectories of brain development, particularly in males with a higher vulnerability to SZ. This knowledge might lead to new strategies of treatment and prevention in SZ.

## Introduction

Obstetric complications (OCs) represent one of the best-replicated environmental risk factors for schizophrenia (SZ) and other psychoses [1], and birth asphyxia, a condition with insufficient oxygen availability to the brain, is among the most consistently implicated [2–5]. Severe birth asphyxia is associated with increased risk of developing SZ (odds ratio = 4.4) [6]. In both patients with SZ and controls, we have previously reported significantly more cases of several severe OCs co-occurring in the presence of birth asphyxia (> 73%) than when birth asphyxia had not been present (< 22%) [7], suggesting birth asphyxia might be a concomitant of complicated pregnancies [8, 9] and of placental pathology [10, 11].

Until early adolescence, total brain volume (TBV) and intracranial volume (ICV) grow in parallel, after which ICV remains static and TBV starts to decrease [12]. The ICV measure is considered a proxy of early brain development, and both neonatal head circumference at birth and head growth in childhood are associated with later intelligence quotient (IQ) [13]. We reported lower premorbid and current IQ in adult patients with SZ and controls who experienced more than one serious OC, wherein birth asphyxia was present in 81% of all such cases of co-occurring OCs [14], suggesting that OCs converging on birth asphyxia may particularly impact brain function early. On an overlapping sample, birth asphyxia was associated with smaller ICV in both adult patients with SZ and controls [5]. Even though birth asphyxia can contribute to the abnormal brain development commonly reported in SZ [15–18], smaller ICV was also found in controls with birth asphyxia [5], which is consistent with studies that show suboptimal head growth and secondary microcephaly after experiencing severe birth asphyxia [19].

A higher polygenic risk score (PRS) for SZ has been reported in patients with SZ and a history of OCs compared to patients without OCs [20], a relationship not found in controls. The interaction was driven by a subset of genomic variants located within or nearby genes highly expressed in the placenta, compared to other tissues, and genes that were upregulated in placentae from complicated pregnancies [20]. PRS derived from this subset of variants are called placental PRS and abbreviates PlacPRS. Conversely, PRS constructed from remaining variants not included in PlacPRS (Non-placental PRSs; NonPlacPRS) did not interact with OCs on SZ-case control status [20].

PlacPRS genes (i.e., PRS based on loci containing genes highly expressed in the placenta and upregulated in placentae from complicated pregnancies) were differentially expressed and specifically upregulated in placentae from male offspring compared to placentae from female offspring [20]. In a follow-up of this work, a negative association between PlacPRS and neonatal ICV was found only in male offspring with a history of OCs, and not in female offspring [21]. Similarly, a negative association between PlacPRS and adult ICV was also found only in adult male patients with SZ [21]. The results suggest that the placental genomic risk factors may play a role in brain development, a higher incidence of SZ in males and may play a critical role in the association between birth asphyxia and risk for SZ development.

While the earlier study showed an association between OCs and PlacPRS depended specifically on serious OCs which likely involved hypoxia, the possible specific role of birth asphyxia in driving the interaction between genomic risk for SZ and OCs has not been specifically analyzed [20, 21]. A failure of replication of such interaction has been reported by a study that used a more limited assessment of OCs and did not assess the exposure to severe OCs linked with birth asphyxia [22]. Using the neonatal head circumference measure from the national Medical Birth Registry of Norway (MBRN) and magnetic resonance imaging (MRI) estimates of adult ICV, the aim of this study was to assess the relationship between PlacPRS and OCs on neonatal and adult head size in a large dataset of patients with SZ and healthy controls. Based on prior data [20, 21], we hypothesized that 1) PRS1 in the presence of birth asphyxia is associated with a higher likelihood of being a patient, an interaction driven by PlacPRS1; and 2) birth asphyxia and PlacPRS1 interaction negatively influence neonatal head circumference and adult ICV, especially in males.

## Materials and methods

### Participants

The study is part of the Thematically Organized Psychosis (TOP) study, which is the main study protocol at the Norwegian Center for Mental Disorders Research (NORMENT; https://www.med.uio.no/norment/english/). Since October 2002, adult patients with SZ were recruited consecutively from psychiatric units (outpatient and inpatient) of public hospitals in the Oslo region. The controls were randomly selected from the national population register in the same catchment area as the patients and asked to participate. All participants gave written informed consent. The study is conducted in accordance with the Helsinki Declaration with approval from the Regional Committee for Medical Research Ethics for South-East Norway (REK sør-øst) and the Norwegian Data Protection Authority (Datatilsynet).

All patients underwent thorough clinical investigation by a trained clinical psychologist and physician. Clinical diagnoses were established using the Structured Clinical Interview for DSM-IV axis 1 disorder (SCID-I) module A-E [23]. The Alcohol Use Disorders Identification Test (AUDIT) [24] and Drug Use Disorders Identification Test (DUDIT) [25] were used to evaluate alcohol and drug use in patients. Controls were interviewed by a trained research assistant and examined with the Primary Care Evaluation of Mental Disorders (Prime-MD) to ensure no current or previous psychiatric disorders [26].

Exclusion criteria for both patients and controls were organic brain disorders (i.e., drug-induced conditions, somatic health conditions, brain damage or head trauma with unconsciousness over 5 min, neurological diseases, autism spectrum disorder). Participants were excluded if IQ < 70, unable to give consent or not proficient in a Scandinavian language. All MRI scans were assessed by a neuroradiologist using a graded scheme [7], and if pathology was detected, the participant was excluded from the study. Additional exclusion criteria for controls were current or previous psychiatric illness, substance use disorders or dependency within the last 6 months and if they or a first-degree relative had a lifetime history of severe psychiatric disorder.

The total sample (*n* = 1303) consisted of patients with a DSM-IV diagnosis within the *schizophrenia spectrum* (SZ, *n* = 433 [schizophrenia, *n* = 327; schizophreniform disorder, *n* = 36; schizoaffective disorder, *n* = 70]) and controls, *n* = 870.

### Obstetric complications

After 16-weeks gestational age, all births in Norway have mandatory reporting to the national Medical Birth Registry of Norway (MBRN). We scored OCs using the validated McNeil–Sjöström scale [27], which includes several hundred complications of potential harm to the fetus/offspring’s central nervous system that are classified according to severity on an ordinal scale from 1 to 6. Participants who had experienced one or more complications of grade 4 through 6 were considered has having a positive history of OCs, consistent with previous work [20, 28–30]. Subjects with complications of grade 3 and below were classified as not having an OC. Small for gestational age (SGA) fetuses were identified in participants, if birth weight was below the 10th percentile for the respective gestational age based on population data from the MBRN. Percentiles for birthweight by gestational age were calculated from 1.8 million births between 1967 and 1998 [31].

The birth asphyxia variable included labor and delivery complications coded AS53 (asphyxia without other signs), AS54 (asphyxia with poor sound), AS55 (asphyxia and discolored amniotic fluid) and AS61 (asphyxia) and neonatal complication coded 7769 (other hypoxic condition, asphyxia) in the MBRN.

### Genotyping

DNA was extracted from blood and saliva samples collected at inclusion. Genotyping was performed on Human Omni Express-24 v.1.1 (Illumina Inc., San Diego, CA, USA) at deCODE Genetics (Reykjavik, Iceland). Pre-imputation quality control was performed using PLINK 1.9 [32]. This involved removal of genetic variants with genotyping rate lower than 95%, Hardy-Weinberg disequilibrium test *p*-value lower than 10^−4^, high rate of Mendel errors in eventual trios or significant (False Discovery Rate < 0.5) batch effects (in case multiple batches were processed simultaneously). Whole individual genotypes were excluded if they had low coverage (< 80%) or high likelihood of contamination (heterozygosity more than 5 standard deviations above the mean). The quality-controlled genotypes were phased using Eagle [33], and missing variants were imputed with MaCH [34, 35] using version 1.1 of the trans-ethnic reference sample put together by the haplotype reference consortium (HRC) [36]. High quality variant sets from the quality control procedure were selected to compute individual’s genetic principal components representing loadings along the 20 first eigenvectors of the pairwise genetic covariance matrix of a sub-sample of unrelated individuals from the HRC panel. Following the quality control and imputation procedure, variants with information score lower than 0.8 or minor allele frequency lower than 0.01 were removed. In addition, individual genotypes imputed with less than 75% confidence were set to missing, the remaining ones were converted to best guess hard allelic dosages.

### Polygenic Risk Score (PRS)

PRS was computed using PRSice-2 [37] version 2.2.11.b (2019-10-16) with default clumping parameters (250 kb clumping window, 0.1 LD r2 threshold using target sample for LD estimation), using summary statistics from the latest and previous GWAS of SZ from the Psychiatric Genomics Consortium (PGC3) [38]. Placental PRSs (PlacPRSs) were calculated, as described in previous work [20, 21], based on the GWAS SNPs marking loci containing genes highly expressed in placenta and differentially expressed in placenta from complicated, compared with normal, pregnancies [20, 21]. The list of placental genes was obtained from Ursini and colleagues [20], and is based on placental gene expression datasets obtained from tissue collected from the fetal/offspring part of the placenta. For the calculation of PlacPRSs and NonPlacPRSs, we therefore selected as “placental” the loci containing genes differentially expressed (*p* < 0.05) in at least four of the eight analyzed datasets of placentae from pregnancies complicated with OCs, and genes with expression in the upper decile both in trophoblast and in villi; the remaining loci are considered as “nonplacental”. This is the same approach used in previous work [20], which provides details of the publicly available datasets used to identify genes highly expressed in placenta and differentially expressed in placentae from pregnancies complicated with preeclampsia and intrauterine growth restriction (IUGR). Odds ratios and index SNPs for PRS calculation were derived from a meta-analysis of Psychiatric Genomics Consortium GWAS datasets. Of note, the PlacPRS loci have similar average effect sizes in the case-control GWAS datasets, compared with the NonPlacPRS loci (*t* = 0.151, *p* = 0.88). In addition, the PlacPRS loci show a significant enrichment for “placenta specific genes” compared with “brain specific genes” (*χ*^*2*^ = 6.53, *p* = 0.005) whose cis-regulated expression in placenta or prenatal cortical brain has been associated with SZ by recent work [39].

### Magnetic resonance imaging (MRI) acquisition and processing

We obtained MRI from three scanners: one 1.5 T scanner (Siemens, Magnetom Sonata; between 2005 and 2011; 116 patients and 187 controls) and two 3 T scanners (General Electric, Signa HDxt [between 2011 and 2015; 52 patients and 264 controls] and General Electric, Discovery MR750 [between 2015 and 2019; 49 patients and 243 controls]).

The 1.5 T Siemens Magnetom Sonata scanner was equipped with an 8-channel head coil and two sagittal T1-weighted magnetization prepared rapid gradient echo (MPRAGE) volumes were acquired with the Siemens tfl3d1_ns pulse sequence: echo time (TE) = 3.93 ms, repetition time (TR) = 2730 ms, inversion time (TI) = 1000 ms, flip angle = 7°, field of view (FOV) = 24 cm, voxel size = 1.33 × 0.94 × 1 mm^3^, 160 sagittal slices.

The 3 T General Electric Signa HDxt was equipped with an 8-channel head coil and T1-weighted 3D Fast Spoiled Gradient Echo (FSPGR) volumes were acquired with scanning parameters: TR/TE = 7.8 ms, TI = 450 ms, flip angle = 12°, FOV = 256 mm, voxel size = 1 × 1 × 1.2 mm^3^, acquisition matrix = 256 × 192.

The 3 T General Electric Discovery MR750 was equipped with a 32-channel head coil and inversion recovery-prepared 3D gradient recalled echo (BRAVO) volumes were acquired with scanning parameters: TR = 8.16 ms, TE = 3.18 ms, TI = 400 ms, flip angle = 12°, FOV = 256 mm, voxel size = 1 × 1 × 1 mm^3^, acquisition matrix = 188 × 256.

T1-weighted MRI volumes were processed in FreeSurfer (v7.0.0) using the cross-sectional SAMSEG processing stream to obtain intracranial volumes (ICV).

To account for the effect of scanner and image acquisition protocols, ComBat harmonization was performed on ICV [40, 41]. Empirical Bayes was used to leverage information across volumes with age, sex, diagnostic group, PlacPRS or NonPlacPRS, 10 genetic principal components, genotyping batches and birth asphyxia entered as variables of interest. Across diagnostic groups, ICV is visualized before and after ComBat harmonization in Supplementary Fig. 1.

### Statistical analyses

#### PRS and case-control status

We used multiple logistic regressions to evaluate the association of each PRS on case–control status in our sample (Diagnosis [Dx] ~ PRS + covariates) and to assess the relationship between PRS and OCs or birth asphyxia with case–control status (Dx ~ PRS + OCs + PRS*OCs + covariates or Dx ~ PRS + birth asphyxia + PRS*birth asphyxia + covariates) in separate models. Age, sex, population stratification (first 10 genetic principal components) and genotyping batch were included as covariates in the model. Similar models were used for PlacPRS, and if significant, NonPlacPRS was also examined. We assessed both the whole sample and European Caucasians in separate analyses, because PRSs are derived from summary statistics of GWASs performed on Caucasians. For these analyses, we report in the main text *t*-statistics and one-tailed *p* values associated with our variables of interest (i.e., PRS*birth asphyxia or PlacPRS*birth asphyxia), based on the directionality of the findings in the previous study [20, 21].

#### PlacPRS and neonatal head circumference

We used multiple regressions to assess the relationship between PlacPRS1 and birth asphyxia on neonatal head circumference (nHC; nHC~ PlacPRS1 + birth asphyxia + PlacPRS1*birth asphyxia + covariates). Age, sex, diagnosis, birthweight, gestational age, first 10 genetic principal components and genotyping batch were included as covariates in the model. nHC values were considered an extreme outlier if they fell outside the third or first quartile, +/− 3 × interquartile range, respectively [42] and subsequentially removed from the data. Because of very low gestational age, two participants (0.24% of sample) were identified as outliers and not included in analysis (see Supplementary Table 1). Models were re-run, stratified by birth asphyxia, sex (female and male only) and diagnosis.

#### PlacPRS and adult intracranial volume

We used multiple regressions to assess the relationship between PlacPRS1 and birth asphyxia on adult ICV (ICV~ PlacPRS1 + birth asphyxia + PlacPRS1*birth asphyxia + covariates). Age, sex, diagnosis, first 10 genetic principal components and genotyping batch were included as covariates in the model. ICV values were considered an extreme outlier if they fell outside the third or first quartile, +/− 3 × interquartile range, respectively [42] and subsequentially removed from the data. Five participants (0.54% of sample) were identified as outliers [42] and removed, as ICV was smaller than 1020 cm^3^ (see Supplementary Table 1 for demographic information on outliers and Supplementary Fig. 2 for plot visualizing method and outliers). Models were re-run, stratified by birth asphyxia, sex (female and male only) and diagnosis.

For both nHC and ICV analyses, we report in the main text *t*-statistics and one-tailed *p*-values associated with our variables of interest (i.e., PlacPRS*birth asphyxia or PlacPRS in stratified models), based on the directionality of the findings in the previous study [20, 21].

Statistical analyses for the relevant methods were performed in SPSS (v28.0) and Python (v3.10).

## Results

### Demographics

Demographic information on the sample is presented separately for males and females in Table 1. We report that significantly more male controls experienced birth asphyxia than female controls. AUDIT scores did not differ between male patients with birth asphyxia (mean [SD]: 8.73 [1.59]) and those without (7.87 [0.56]; F = 0.26, *p* = 0.612) or female patients with birth asphyxia (5.31 [3.37]) and those without (4.69 [2.85]; F = 1.09, *p* = 0.298). DUDIT scores did not differ between male patients with birth asphyxia (mean [SD]: 4.38 [7.06]) and those without (4.96 [8.02]; F = 0.10, *p* = 0.752) or female patients with birth asphyxia (2.06 [4.98]) and those without (3.47 [7.94]; F = 0.43, *p* = 0.514).

**Table 1:** Demographic information presented separately for males and females.

Males									Pairwise comparisons	
	HC (*n* = 467)			SZ (*n* = 254)			Main effect Group		SZ vs HC	
	ASPH-	ASPH+	*F/*χ^*2*^ (df)	ASPH-	ASPH+	*F/*χ^*2*^ (df)	*F/*χ^*2*^ (df)	*p*	Mean differences (SE)	*p*
Sex, No. Males (%)	393 (84%)	74 (16%)	4.98 (1, 870)^a^*	226 (89%)	28 (11%)	0.57 (1, 433)	3.15 (1, 721)	0.076 ^b^		(HC > SZ)
Ancestry, No. non-European (%)	5 (1%)	1 (1%)	0.00 (1, 467)	31 (14%)	4 (14%)	0.00 (1, 254)	47.89 (1, 721)	< 0.001		(SZ > HC)
Handedness, No. NR (%)	46 (12%)	6 (8%)	0.85 (1, 464)	31 (15%)	2 (8%)	0.92 (1, 230)	1.41 (1, 694)	0.235		-
Age, mean years (SD)	31.71 (7.88)	30.82 (7.17)	0.80 (1, 467)	27.00 (6.55)	28.14 (6.68)	0.76 (1, 254)	59.87 (1, 721)	< 0.001	−4.45 (0.58)	< 0.001
Education, years (SD)	14.51 (2.32)	14.16 (2.14)	1.46 (1, 464)	12.17 (2.56)	11.77 (1.68)	0.61 (1, 231)	151.33 (1, 695)	< 0.001	−2.33 (0.19)	< 0.001
Adult weight, kg (SD)	83.73 (12.57)	83.49 (14.14)	0.02 (1, 365)	84.78 (18.71)	89.55 (18.92)	1.56 (1, 242)	1.60 (1, 607)	0.207	1.62 (1.28)	0.207
Adult height, cm (SD)	182.80 (5.91)	180.69 (6.04)	6.39 (1, 367)*	180.48 (7.06)	180.07 (7.63)	0.08 (1, 244)	14.31 (1, 611)	< 0.001	−2.02 (0.53)	< 0.001
Adult ICV, cm^3^ (SD)	1654.71 (114.35)	1632.20 (109.57)	2.08 (1, 396)	1624.09 (122.06)	1692.82 (121.56)	2.93 (1, 133)	3.53 (1, 529)	0.061	−21.88 (11.64)	0.061
Gestational age, weeks (SD)	39.69 (1.86)	39.81 (2.75)	0.21 (1, 440)	39.49 (2.43)	38.62 (4.38)	2.44 (1, 237)	2.79 (1, 677)	0.095	−0.31 (0.18)	0.095
Birth weight, grams (SD)	3595.46 (555.48)	3555.68 (746.94)	0.28 (1, 466)	3545.67 (548.22)	3287.86 (896.24)	4.67 (1,253)*	2.42 (1, 719)	0.120	−72.01 (46.31)	0.120
SGA, No. SGA (%)	48 (13%)	4 (6%)	2.74 (1, 439)	19 (9%)	5 (19%)	2.63 (1, 236)	0.43 (1, 675)	0.511		-
Neonatal head circumference, cm (SD)	35.54 (1.57)	35.35 (1.50)	0.55 (1, 275)	35.41 (1.44)	35.87 (1.64)	1.37 (1, 175)	0.17 (1, 450)	0.685	−0.06 (0.15)	0.685

Females									Pairwise comparisons	
	HC (*n* = 403)			SZ (*n* = 179)			Main effect Group		SZ vs HC	
	ASPH-	ASPH+	*F/*χ^*2*^ (df)	ASPH-	ASPH+	*F/*χ^*2*^ (df)	*F/*χ^*2*^ (df)	*p*	Mean differences (SE)	*p*
Sex, No. Females	360 (89%)	43 (11%)	4.98 (1, 870)^a^*	155 (87%)	24 (13%)	0.57 (1, 433)	0.91 (1, 582)	0.340		-
Ancestry, No. non-European (%)	8 (2%)	0 (0%)	0.98 (1, 402)	14 (9%)	4 (17%)	1.34 (1,179)	18.85 (1, 581)	< 0.001		(SZ > HC)
Handedness, No. NR (%)	35 (10%)	3 (7%)	0.30 (1, 400)	17 (12%)	2 (11%)	0.01 (1, 159)	0.75 (1, 559)	0.388		-
Age, mean years (SD)	30.97 (7.99)	28.70 (6.60)	3.22 (1, 403)	27.17 (7.21)	26.42 (6.90)	0.23 (1, 179)	28.19 (1, 582)	< 0.001	−3.65 (0.69)	< 0.001
Education, years (SD)	14.58 (2.09)	14.60 (2.03)	0.00 (1, 400)	12.44 (2.74)	11.42 (1.61)	2.49 (1, 159)	115.39 (1, 559)	< 0.001	−2.27 (0.21)	< 0.001
Adult weight, kg (SD)	66.47 (10.69)	65.29 (12.54)	0.34 (1, 290)	73.42 (18.13)	65.83 (11.47)	3.94 (1, 170)*	20.62 (1, 460)	< 0.001	6.02 (1.33)	< 0.001
Adult height, cm (SD)	168.13 (6.19)	166.70 (4.47)	1.65 (1, 292)	167.40 (5.72)	167.42 (5.71)	0.00 (1, 173)	0.98 (1, 465)	0.323	−0.56 (0.57)	0.323
Adult ICV, cm^3^ (SD)	1455.92 (94.73)	1437.40 (84.81)	1.27 (1, 298)	1429.52 (118.51)	1446.44 (104.75)	0.22 (1, 84)	3.14 (1, 382)	0.077	−21.68 (12.23)	0.077
Gestational age, weeks (SD)	40.02 (1.57)	40.41 (1.61)	2.21 (1, 384)	39.89 (1.85)	39.82 (2.72)	0.03 (1, 167)	1.37 (1, 551)	0.242	−0.19 (0.16)	0.242
Birth weight, grams (SD)	3454.22 (497.64)	3383.26 (83.86)	0.79 (1, 403)	3469.28 (478.14)	3334.58 (842.66)	1.30 (1, 179)	0.10 (1, 582)	0.921	4.57 (45.81)	0.921
SGA, No. SGA (%)	38 (11%)	6 (14%)	0.37 (1, 384)	14 (10%)	5 (23%)	3.24 (1, 167)	0.00 (1, 675)	0.978		-
Neonatal head circumference, cm (SD)	34.96 (1.31)	34.91 (1.42)	0.04 (1, 257)	34.86 (1.20)	34.81 (1.64)	0.02 (1, 122)	0.50 (1, 379)	0.481	−0.10 (0.14)	0.481

*HC* Healthy controls, *SZ* Patients with schizophrenia, *NR* Non-right-handed, *SD* Standard deviation, *df* degrees of freedom, *SE* Standard error of mean, *ICV* Intracranial volume, *SGA* Small for gestational age.

^a^Significantly more males with ASPH than females with ASPH.

^b^Significantly more HC males with ASPH than SZ males with ASPH using the One-sided Fisher’s Exact Test = 0.047.

*p < 0.05.

### Obstetric complications

OCs scored greater than 3 using the McNeil–Sjöström scale are listed in Supplementary Table 2. The number of individuals experiencing a serious OC (No./total sample [%]: 204/433 [47%] patients and 393/870 [45%] controls) or birth asphyxia (52/433 [12%] patients and 117/870 [13%] controls) was not significantly different (χ^2^ = 0.44, *p* = 0.508 and χ^2^ = 0.53, *p* = 0.466, respectively) between groups. Significantly more OCs co-occurred in the presence of birth asphyxia (No./total sample [%]: 151/169 [89%]) than when birth asphyxia was not present (428/1134 [38%]; χ^2^ = 158.65, *p* < 0.001). The incidence of SGA was similar between the groups (patients, 43/403 [11%]; healthy controls, 96/823 [12%]; χ^2^ = 0.27, *p* = 0.606 and was not higher in males (76/675 [11%]) compared to females (63/551 [11%]; χ^2^ = 0.01, *p* = 0.924; see Table 1).

### PRS1 on case-control status

**Fig. 1:**
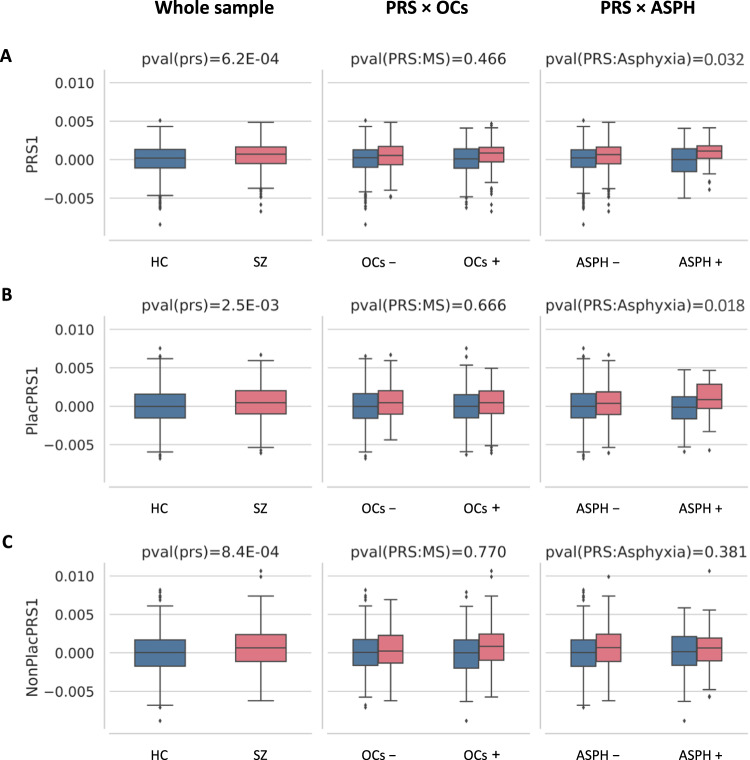
PRS1, PlacPRS1, NonPlacPRS1 and a history of obstetric complications (OCs) or birth asphyxia (ASPH) in the TOP sample of schizophrenia (SZ) patients and healthy controls (HC). **A** Association between genomic risk score (PRS1), constructed from alleles showing significant association with SZ (GWAS *p* < 5E-08) and case-control status. Interaction between PRS1 and OCs (scored > 3 with the McNeil–Sjöström scale, MS) on case–control status. Interaction between PRS1 and ASPH on case–control status. **B** Association between placental risk score (PlacPRS1), constructed from alleles showing significant association with SZ (GWAS *p* < 5E-08) and genes highly and differentially expressed in placentae, and case–control status. Interaction between PlacPRS1 and OCs (scored > 3 with the McNeil–Sjöström scale, MS) on case–control status. Interaction between PlacPRS1 and ASPH on case–control status. **C** Association between non-placental risk score (NonPlacPRS1), constructed from alleles showing significant association with SZ (GWAS *p* < 5E-08) and genes not expressed in placenta, and case–control status. Interaction between NonPlacPRS1 and OCs (scored > 3 with the McNeil–Sjöström scale, MS) on case–control status. Interaction between NonPlacPRS1 and ASPH on case–control status. One-tailed *p* values are reported for both PRS1*birth asphyxia and PlacPRS1*birth asphyxia in the Figure, based on the directionality of the findings in previous study [20, 21]. See Table 2 and main text for detailed statistics.

The PRS constructed from alleles showing the most significant GWAS association with SZ (*p* < 5E-08; PRS1) was positively associated with case–control status (*n* = 1303, *t* = 3.42, *p* < 0.001), confirming that patients had greater polygenic risk for SZ than controls. These results were similar for PlacPRS1 (*t* = 3.03, *p* = < 0.001), and NonPlacPRS1 (*t* = 3.34, *p* = < 0.001) on case–control status (Fig. 1 and Table 2). We did not find a significant interaction between PRS1 or PlacPRS1 and OCs (Table 2), but we did find a significant interaction between PRS1 and birth asphyxia on case–control status (*t* = 1.85, *p* = 0.032). A significant interaction was observed between PlacPRS1 and birth asphyxia on case–control status (*t* = 2.10, *p* = 0.018), suggesting that birth asphyxia may represent a common factor underlying OCs specifically interacting with the placental component of genomic risk for SZ. We did not find a significant interaction between NonPlacPRS1 and birth asphyxia on case-control status (*t* = −0.88, *p* = 0.381; Fig. 1 and Table 2). Here we focus on PRS1, since these risk scores are based on the most reliable and tractable GWAS findings and previous evidence of their interaction with serious OCs [20, 21]. Results of PGC3 European Caucasians only for PRS1 are presented in Supplementary Table 3.

**Table 2:** Multiple logistic regression variables of interest for the whole sample.

Diagnosis															
	Whole sample (n = 1303)			ASPH absence (n = 1134)			ASPH presence (n = 169)			PRS*ASPH		PRS*OCs (no ASPH)		PRS*OCs	
	*t*	*p*	*R*^*2*^	*t*	*p*	*R*^*2*^	*t*	*p*	*R*^*2*^	*t*	*p*	*t*	*p*	*t*	*p*
PRS1	3.42	6.20E-04	0.0073	2.56	5.21E-3	0.0046	2.63	4.29E-3	0.0365	1.85	**0.032**	−0.14	0.886	0.73	0.466
PlacPRS1	3.03	2.50E-03	0.0056	2.18	1.48E-2	0.0033	2.38	8.61E-3	0.0292	2.1	**0.018**	−1.45	0.148	−0.43	0.666
NonPlacPRS1	3.34	8.40E-04	0.0068	3.42	3.07E-4	0.0082	0.29	3.85E-1	0.0004	−0.88	0.381	0.74	0.457	0.29	0.770

We found a relationship between each PRS1 and case-control status (column 3). Columns 2–10 report the statistics (*t* and *p*-values and *R*^*2*^: Nagelkerke *R*^*2*^, goodness-of-fit) for the relationship between PRS1 and case-control status in the whole sample (columns 2–4) and in individuals without (columns 5–7) and with (columns 8–10) a history of birth asphyxia. Columns 11–12 report the statistics for the interaction between each PRS1 and a history of birth asphyxia (ASPH) on case-control status. Columns 13–14 shows the statistics for the interaction between PRS1 and OCs in individuals with OCs but without birth asphyxia, those 169 individuals were removed from the analysis. Columns 15–16 contain the statistics for the interaction between PRS1 and a history of obstetric complications (OCs) on case-control status. All statistics were generated using multiple logistic regression, adjusting for population stratification (10 PCs), genotype batch, age and sex. Bolded numbers represent significant *p*-values.

### PlacPRS and neonatal head circumference (nHC)

**Fig. 2:**
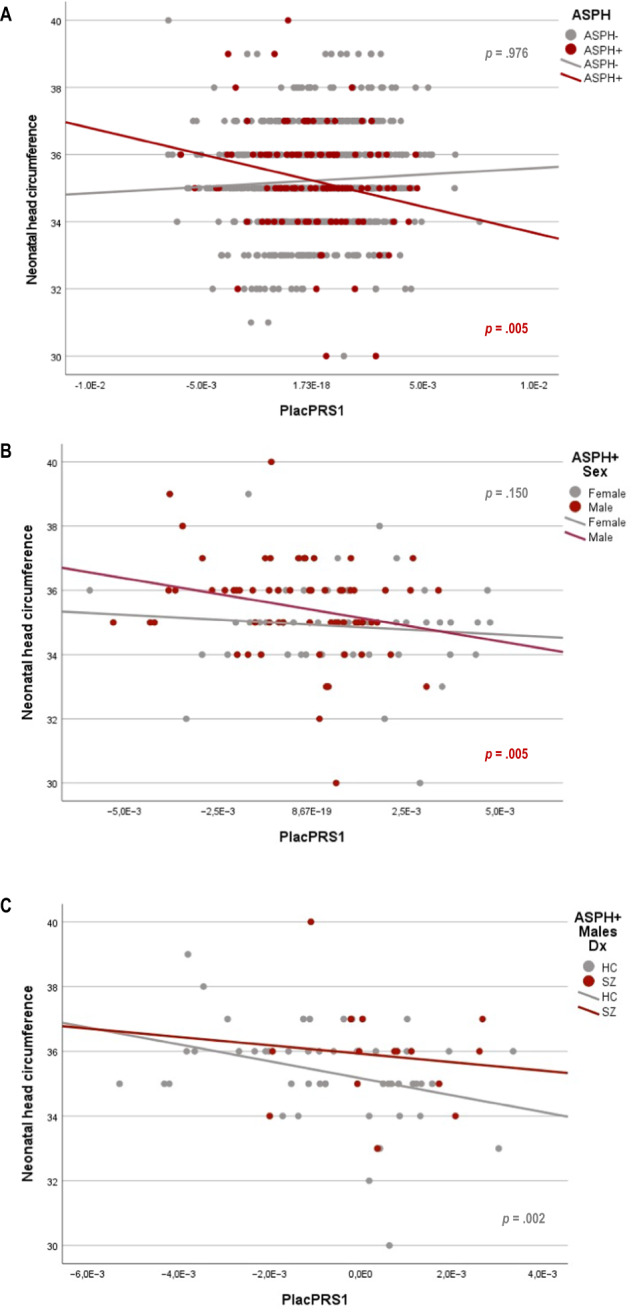
Placental genomic risk for schizophrenia and neonatal head circumference in centimeters (cm; *n* = 829). **A**. Scatterplot of the relationship of neonatal head circumference with placental genomic risk scores for schizophrenia, constructed from alleles showing the most significant association with schizophrenia (GWAS *p* < 5 × 10^−8^; PlacPRS1) within loci containing genes highly expressed in placenta and differentially expressed in placentae from complicated compared with normal pregnancies. The figure shows the relationship between neonatal head circumference and PlacPRS1 in participants who experienced birth asphyxia (ASPH + ; *n* = 114; red dots) and participants who did not (ASPH-; *n* = 715; gray dots), with the *p*-value in respective colors for each group: Only ASPH+ participants show a negative relationship. **B** Sex-related differences in the relationship between PlacPRS1 and neonatal head circumference. Scatterplot of the relationship of neonatal head circumference with PlacPRS1 in females with ASPH (*n* = 51; gray dots) and males with ASPH (*n* = 63; red dots), with *p*-values in respective colors for each group. Only male participants show a negative relationship. **C** Scatterplot of the relationship of neonatal head circumference with PlacPRS1 in male controls with ASPH (*n* = 48; gray dots) and male patients with ASPH (*n* = 15; red dots), with *p*-values in respective colors for each group. Both ASPH+ male controls and patients show a negative relationship, although in patients’ statistical significance could not be determined/tested due to low n. HC Healthy controls, SZ Patients with schizophrenia. One-tailed *p*-values are reported in the figure, based on the directionality of the findings in previous study [20, 21]. See main text and Supplementary Table 5 for detailed statistics.

We found a significant interaction between PlacPRS1 and birth asphyxia on nHC in the whole sample (*t* = −2.43, *p* = 0.008; Supplementary Table 4), such that higher placental genomic risk for SZ was associated with lower nHC in those with birth asphyxia (Fig. 2A). This relationship was significant in males (*t* = −2.71, *p* = 0.005), but not for females (*t* = −1.06, *p* = 0.150; Supplementary Table 5 and Fig. 2B). Further, the association was significant in male controls with birth asphyxia (*t* = −3.24, *p* = 0.002), but there were too few male patients with birth asphyxia to estimate the model (Supplementary Table 5 and Fig. 2C).

### PlacPRS and adult intracranial volume (ICV)

**Fig. 3:**
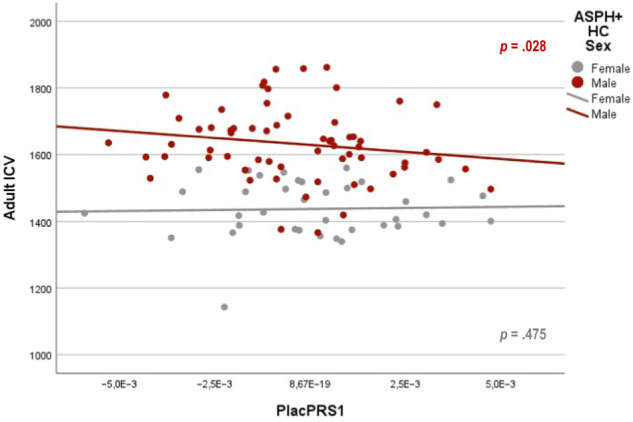
Placental genomic risk for schizophrenia and adult intracranial volume (ICV) in cubic centimeters (cm^3^; *n* = 100). Scatterplot of the relationship of adult ICV with placental genomic risk scores for schizophrenia, constructed from alleles showing the most significant association with schizophrenia (GWAS *p* < 5 × 10^−8^; PlacPRS1) within loci containing genes highly expressed in placenta and differentially expressed in placentae from complicated compared with normal pregnancies. Scatterplot of the relationship of adult ICV with PlacPRS1 in control females with ASPH (*n* = 37; gray dots) and control males with ASPH (*n* = 63; red dots), with *p*-values in respective colors for each group. One-tailed *p*-values are reported in the figure, based on the directionality of the findings in previous study [20, 21]. See main text and Supplementary Table 7 for detailed statistics.

We did not find a significant interaction between PlacPRS1 and birth asphyxia on adult ICV in the whole sample (*t* = −0.21, *p* = 0.418; Supplementary Table 6). However, we did find a significant negative association between PlacPRS1 and adult ICV (*t* = −1.97, *p* = 0.028) in male controls with birth asphyxia, but not in female controls with birth asphyxia (*t* = −0.06, *p* = 0.475; Supplementary Table 7 and Fig. 3). There were no significant associations when adding gestational age and birth weight to the model. In the patient group, there were too few patients with SZ and birth asphyxia to perform sex-stratified analyses.

## Discussion

We provide evidence of a gene × environment interaction related to genomic risk for SZ and the serious obstetric complication (OC) birth asphyxia, which is in line with principal findings from the larger study of Ursini and colleagues [20]. We show that PRS constructed from loci harboring genes abundantly expressed in placenta (i.e., PlacPRS) appears to capture this interaction. We likely confirm that PlacPRS is associated with brain size at birth, at least to the extent that it corresponds to neonatal head circumference (nHC), especially in males with a history of asphyxia [21]. Our results suggest that, in males with a history of birth asphyxia, genomic variants related to the placental response to hypoxia, metabolic and cellular stress (i.e., the PlacPRS variants) may underlie key features of brain development and related developmental neuropathologies.

Common genomic variants and environmental risk factors related to SZ are frequent in a population and in part shared with other psychiatric disorders, such as bipolar disorder, major depressive disorder, autism spectrum disorder [43]. It is therefore possible that our comparison between patients with SZ and controls without any psychiatric disorders also highlights the effect of risk factors that are not unique to SZ and that can affect neurodevelopmental alterations, which can be common to other psychiatric disorders and contribute to neurodevelopmental variation in the general population.

However, in evolutionary genetics, the Conrad Waddington model posits that adverse environmental events and epigenetic changes diminish the capacity to buffer a normal developmental trajectory, which can lead to the decanalization (i.e., derailment) of a phenotype and increased variability of a trait in a population [44, 45]. Under this model, we suggest that the interplay between the neuropathological insult of birth asphyxia and genomic variants related to the inability of male placentae to tolerate hypoxic stress may underlie key features in the epidemiology of SZ. Specifically, birth asphyxia and higher placental genomic risk decanalize male brain development, and SZ, a behavioral phenotype, is associated with the increased variance associated with this decanalization of brain development.

There was evidence of a negative association between PlacPRS and adult ICV still being present in male controls with birth asphyxia, which suggests that placental biology related to SZ risk and birth asphyxia influence brain development longitudinally and can affect a phenotype that is associated with susceptibility to the disorder but not necessarily the transition to it. It is possible that the male patients, who experience birth asphyxia, have a more severe neurodevelopmental course, which would preclude inclusion into the study [46]. Because of the smaller sample size, the data is seemingly not powered enough to address whether the relationship is or is not present for males with SZ. Nevertheless, the negative association in both male patients and controls is in accordance with previous research, which shows that placental pathology is associated with altered neurodevelopment particularly in males [47–49]. Hypoxia-related alterations (e.g., mitochondrial dysregulation) in in vitro cellular models suggest decreased physiological resilience in astrocytes to hypoxia and resulting cellular stress in SZ [50, 51]. Decreased or impaired cellular resilience can render glial cells, such as astrocytes, unable to perform their supportive, neuroprotective and/or neurorestorative activities [52]. Thus, the inability of the developing male brain to compensate for hypoxic stress may be a result, at least in part, of pathophysiological alterations in brain cells in a complex constellation of gene × environment interactions involving the developing brain and the placenta.

Our results do not clearly indicate the molecular mechanisms through which placental genomic risk and birth asphyxia affect altered neurodevelopmental trajectories and risk for SZ. However, they are consistent with the possibility that placental genes play a role and expand the current view on the main etiopathogenetic hypothesis for SZ [53], which assumes that genomic variants associated with risk disrupt brain development and function. In a recent study by Ursini and colleagues [39], they used placental tissue to identify placental genes with a causal role for SZ and performed analogous analysis in the fetal brain. They identified 139, many being sex-biased, placental specific SZ risk genes with expression in both the placenta and brain. Candidate molecular mechanisms implicate nutrient-sensing capabilities and trophoblast invasiveness of the placenta, which suggests genomic risk factors might lead to alteration in brain development via the placenta [39].

A possible limitation of our study is represented by the fact that we analyzed a sample where OCs and birth asphyxia do not show an association with SZ. This is in apparent contrast with studies that have shown a consistent association of many severe OCs and birth asphyxia with risk for psychosis and SZ [1]. This inconsistency may be affected by power issues, given our relatively small sample size, and is common to other studies [2, 5, 7, 14, 20, 54, 55] where the inclusion of only individuals with certain information about the presence or absence of OCs may result in a sample with a higher frequency of OCs and birth asphyxia compared with the general population.

It is important to note that the statistical relationship for the PRS1 and PlacPRS1 in the presence of birth asphyxia is weak, with Nagelkerke *R*^2^ = 0.037 and 0.029, respectively. However, consistent with previous work [20], the liability of SZ explained by PRS1 and PlacPRS1 is respectively 7.93 and 8.85 times higher in presence of asphyxia, compared with when asphyxia is absent. In addition, and again consistent with previous work [21], PlacPRS1 is associated with early neurodevelopmental outcomes, in presence of birth asphyxia. While further replication is needed, we believe our findings support previous research [20, 21] indicating that the most significant genetic variants associated with SZ may converge on a developmental trajectory sensitive to serious OCs specifically related to asphyxia, and particularly relevant in males [56].

In comparison to previous PRS and OCs studies in SZ [20, 22], the use of birth registry data is unique to this study, and the use of the McNeil–Sjöström scale for more detailed assessment of OCs is similar to Ursini and colleagues [20] but different from Vassos and colleagues [22]. Antenatal factors related to maternal infections, smoking and drug use and postpartum factors such as maternal stress and depression are important factors when interpreting the data; however, this information was unavailable. The wide definition and lack of details in the reporting of birth asphyxia to the MBRN are also limitations in this study. The presence of birth asphyxia in the MBRN provides a record of a clinically relevant deficiency in brain oxygenation of affected newborns, but it does not provide information on the severity, treatments administered or results of neonatal MRI examinations. Participants with pathological findings on MRI examination, neurological disorders or intellectual disability were not included in the study, and hence it is possible that the study design does not fully capture the severe end of the hypoxic spectrum in patients. The less significant associations in male patients might be a result of the smaller sample size, compared with controls, which provides less power to detect small and interactive effects in the subsample of patients with birth asphyxia, and of the study design, excluding patients with organic brain disorders (i.e., somatic health conditions, traumatic brain injury, neurological diseases, autism spectrum disorder) and IQ < 70.

Another limitation of this study is represented by the fact that, although PlacPRSs are genomic predictors derived by placental gene-expression data, we cannot exclude that part of the effect of the PlacPRS is related to a more direct effect in the brain of the PlacPRS genes. However, the absence of a significant interaction of NonPlacPRSs with birth asphyxia is consistent with the possibility that genomic predictors based on placental gene expression may have a more specific role in affecting early trajectories of brain development and risk for SZ in individuals with a history of birth asphyxia. Future work may help to refine the calculation of placental genomic predictors by leveraging findings related to genes whose cis-regulated expression in placenta, and not in brain, is associated with SZ [39].

## Conclusion

The significant interaction between birth asphyxia and PlacPRS on case-control status revealed that a higher PlacPRS in the presence of birth asphyxia was associated with a higher likelihood of being a patient. Further, a negative association between PlacPRS and neonatal and adult head size was found in males with history of birth asphyxia. These findings suggest that placental pathophysiology and birth asphyxia play a role in affecting early and late trajectories of brain development, potentially linked with a higher vulnerability to SZ in males. New strategies of treatment and prevention may benefit from understanding how placental genomic risk for SZ and birth asphyxia can contribute to the decanalization of brain development.

### Supplementary information

SUPPLEMENTAL MATERIAL

Supplementary Figures 1 and 2

## Data Availability

The datasets used and/or analyzed during the current study are available from the corresponding author on reasonable request. The data are not publicly available due to privacy or ethical restrictions. Supplementary information is available at TP’s website.
