# Supplementary material for: The impact of placental genomic risk for schizophrenia and birth asphyxia on brain development
Source: Transl Psychiatry. 2023 Nov 8;13:343. doi: 10.1038/s41398-023-02639-4 (PMC10632427; doi:10.1038/s41398-023-02639-4)
Supplement: Supplementary file 2 — Supplementary Figures 1 and 2 [file 41398_2023_2639_MOESM2_ESM.docx]

**The impact of placental genomic risk for schizophrenia and birth asphyxia on brain development**

Laura A. Wortinger PhD * ^1, 2^, Alexey A. Shadrin PhD ^2, 3, 4^, Attila Szabo PhD ^2, 3, 4^, Stener Nerland MSc ^1, 2^, Runar Elle Smelror PhD ^1, 2^, Kjetil Nordbø Jørgensen PhD ^1, 5^, Claudia Barth PhD ^1, 2^, Dimitrios Andreou MD PhD ^1, 2, 6^, Marianne Thoresen MD PhD ^7, 8^, Ole A. Andreassen MD PhD ^2, 3, 4^, Srdjan Djurovic PhD ^4, 9, 10^, Gianluca Ursini MD PhD ^11, 12^ and Ingrid Agartz MD PhD ^1, 2, 4, 6^

^1^Department of Psychiatric Research, Diakonhjemmet Hospital, Oslo, Norway

^2^ NORMENT, Institute of Clinical Medicine, University of Oslo, Oslo, Norway

^3^NORMENT, Division of Mental Health and Addiction, Oslo University Hospital, Oslo, Norway

^4^KG Jebsen Centre for Neurodevelopmental Disorders, University of Oslo, Oslo, Norway

^5^Department of Psychiatry, Telemark Hospital, Skien, Norway

^6^Centre for Psychiatry Research, Department of Clinical Neuroscience, Karolinska Institutet and Stockholm Health Care Services, Stockholm County Council, Stockholm, Sweden

^7^Department of Physiology, Institute of Basic Medical Sciences, University of Oslo, Oslo, Norway

^8^Neonatal Neuroscience, Translational Health Sciences, University of Bristol, Bristol, United Kingdom

^9^Department of Medical Genetics, Oslo University Hospital, Oslo, Norway

^10^NORMENT, Department of Clinical Science, University of Bergen, Bergen, Norway

^11^Lieber Institute for Brain Development, Johns Hopkins Medical Campus, Baltimore, MD, USA

^12^Department of Psychiatry and Behavioral Sciences, Johns Hopkins University School of Medicine, Baltimore, MD, USA

* To whom correspondence should be addressed: Dr. Laura A. Wortinger, Department of Psychiatric Research, Diakonhjemmet Hospital, Postbox 23 Vinderen, 0319, Oslo, Norway; telephone: +4792282220; e-mail: [l.a.w.bakke@medisin.uio.no](mailto:l.a.w.bakke@medisin.uio.no)

Supplementary Figure 1. ComBat harmonization

Supplementary Figure 2. Boxplot visualizing nHC and adult ICV outliers

**Supplementary Figure 1.** Intracranial volume before and after ComBat harmonization. ComBat harmonization was performed on adult ICV (1, 2) to account for the effect of scanner and image acquisition protocols.


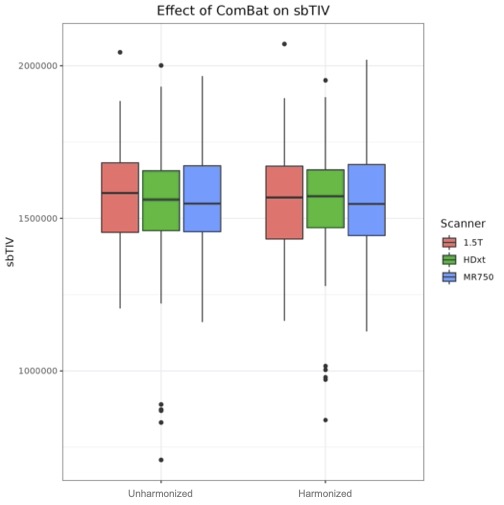


**Supplementary Figure 2.** Boxplot visualizing (**A**) neonatal head circumference (nHC; cm) and (**B**) adult intracranial volume (ICV; cm^3^) outliers. nHC and ICV values were considered an extreme outlier if they fell outside the third or first quartile, +/- 3 × interquartile range, respectively (3) and subsequentially removed from the data.

**
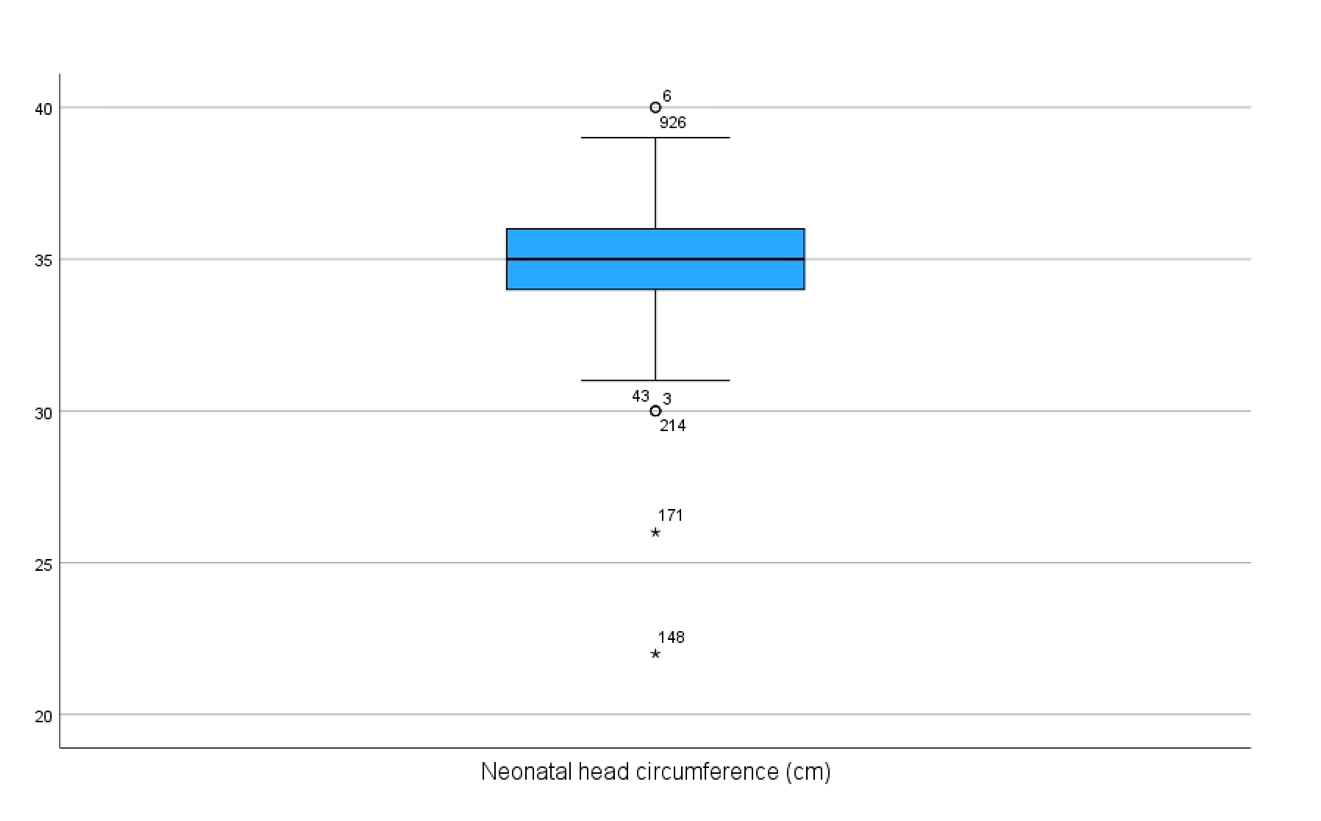
**

**A**

**B**

**
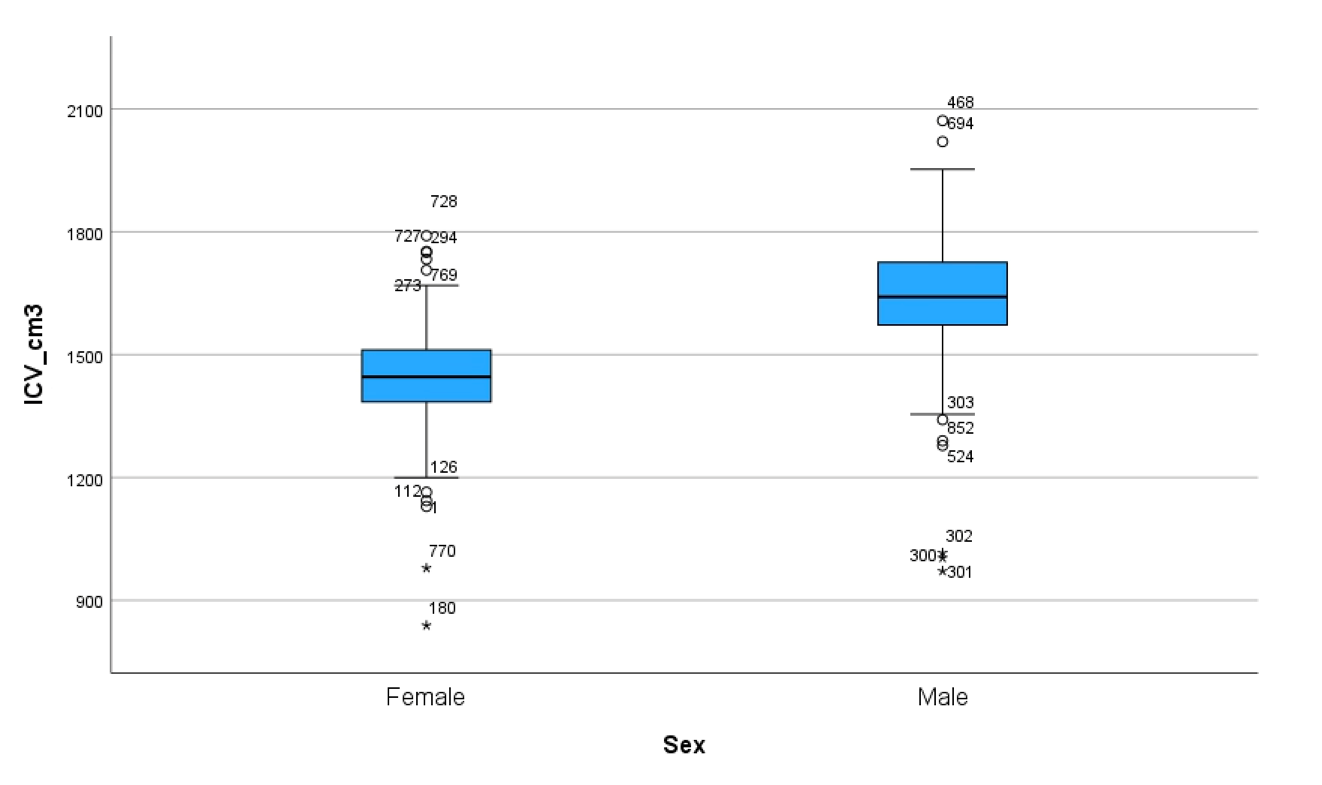
**

**References**

1. Fortin JP, Cullen N, Sheline YI, Taylor WD, Aselcioglu I, Cook PA, Adams P, Cooper C, Fava M, McGrath PJ, McInnis M, Phillips ML, Trivedi MH, Weissman MM, Shinohara RT. Harmonization of cortical thickness measurements across scanners and sites. Neuroimage. 2018;167:104-120.

2. Johnson WE, Li C, Rabinovic A. Adjusting batch effects in microarray expression data using empirical Bayes methods. Biostatistics. 2007;8:118-127.

3. Tukey JW: Exploratory data analysis. Reading, MA, Addison-Wesley Pub. Co.; 1977.
